# Further Hierarchies of Loss: Tracking Relationality in Pregnancy Loss Experiences

**DOI:** 10.1177/00302228231182273

**Published:** 2023-06-07

**Authors:** Aimee L. Middlemiss, Susie Kilshaw

**Affiliations:** 1 Wellcome Centre for Cultures and Environments of Health, 215734 University of Exeter, Exeter, UK; 2 Department of Anthropology, 4919 University College London, London, UK

**Keywords:** grief, pregnancy loss, miscarriage, abortion, hierarchy, politics of death

## Abstract

The article extends Robson and Walter’s concept of hierarchies of loss by describing further factors which afford differential social legitimacy to death-related losses. Drawing on our separate research with women in England who have experienced pre-viability pregnancy loss through different types of miscarriage and termination for foetal anomaly, we note that closeness of relationship to the object of loss does hierarchise pregnancy loss. However, other relational elements are also implicated, including ontological positions on what it was which was lost, in relation to other individually and socially experienced losses. Hierarchies are both imposed and agentially used by those who are implicated. This wider analysis extends the concept of hierarchies of loss so it can include experiences which do and do not involve grief and bereavement, and experiences of social recognition alongside those where loss is disenfranchised, marginalised, or ungrievable.

## Introduction

In the paper which introduced the concept of ‘hierarchies of loss’, Robson and Walter (2013) speculated that hierarchies related to social recognition of loss might be found in perinatal death, abortion, and miscarriage. In this paper, drawing on our separate qualitative social science research with women in England who have experienced pre-viability pregnancy loss through miscarriage and termination for foetal anomaly, we find that there are indeed hierarchies of loss based on some persons being afforded more social legitimacy in their reaction to loss. However, we also find that the factors which hierarchise loss are more complex and diverse than simply the closeness of relationship to the deceased. We argue the analytic possibilities of the concept of hierarchies of loss can usefully include attention to other relational elements. These include ontological positions on what it was which was lost, in relation to other individually and socially experienced losses, and the social and political consequences of such losses. Tracing these wider aspects of hierarchies of loss allows for a more nuanced and complex understanding of loss in which experiences which fall outside assumed norms can be included and examined. The breadth of possibility in invoking hierarchies of loss also makes visible opportunities for agency on the part of those implicated in the hierarchies. Analysis of pre-viability pregnancy loss experiences therefore extends the concept of hierarchies of loss to provide further ways of tracing the politics and relationality of loss.

### Social Contexts of Loss, Grief and Bereavement

In the aftermath of a death or loss, effects are experienced on a personal, individual register and in relation to structures and discourses of social life. This social context of grief and loss can legitimate sadness or mourning and offer normative social processes through which grief can be expressed or affirmed. It can also classify some losses as inappropriate foci of grief or mourning, as expressed by Doka’s concept of ‘disenfranchised grief’ (1989), in which there is a conflict between the emotional or internal experience of a grieving person and the social acceptability of their expression of this grief. For example, certain relationships, losses, or grieving persons may not be socially recognised and this may have consequences for those who grieve (Doka, 1989; 2002). Since Doka first posited the possibility of disenfranchised grief after miscarriage and abortion, events of pregnancy loss and perinatal loss in several contexts have been analysed as producing disenfranchised grief (Cassidy, 2021; Faro, 2020; Hazen, 2003; Lafarge et al., 2019; Lang et al., 2011). The concept dovetails with other tropes found in pregnancy loss literature such as silence and the silencing of those who experience miscarriage (Kilshaw & Borg, 2020; van der Sijpt, 2017), and the absence of cultural scripts to understand or express grief at the end of a pregnancy (Frost et al., 2007; Layne, 2003).

However, Robson and Walter (2013), working in a British empirical context, argue that the social acceptability of grief and mourning is more nuanced that the binary of a fully enfranchised, acceptable grief versus a fully disenfranchised, unacceptable grief. They propose a scalar, non-binary concept of ‘hierarchies of loss’ in which social norms define which deaths it is legitimate to grieve, to what degree, in relation to those who are bereaved. In this model, experiences of disenfranchisement come about in certain specific circumstances as a result of hierarchies of loss when ‘the hierarchy indicates someone should grieve less than they actually do’ (Robson & Walter, 2013, p. 101). Hierarchies of loss seek to explain the social norms and disjunctions which may then result in disenfranchised grief for some people. In Robson and Walter’s research, the social acceptability of grief intensity and duration was found to be derived from normative assessments of the closeness of relationship between the deceased and those who experienced the loss, resulting in hierarchies of loss in which some people were considered to have suffered a more serious or impactful loss than others. For example, those who were biologically related to the deceased person, and those who were closer in kinship terms, were considered to have suffered a more serious loss than those in a professional relationship with the deceased.

Previous literature, therefore, has noted that not all loss and death is treated as socially legitimate, that some who have experiences of loss and death are completely excluded from social recognition and others are ranked and hierarchised according to the perceived degree to which they are affected by the event. One factor affecting this ranking is social closeness to the object of loss or death. In this paper, we draw on wider literature about the politics of loss to identify further factors which produce and express hierarchies of loss. One such factor is the perceived value and ontological status of the object of loss itself. In the case of pregnancy loss literature, the idea that some losses are more impactful or serious that others has been noted since Lovell’s early work on late miscarriage, stillbirth and perinatal loss (Lovell, 1983). Lovell used the term ‘hierarchies of sadness’ to discuss how medical professionals considered the gestational age of the foetal being or baby to produce different degrees of loss, with a neonatal death constructed as ‘worse’ than a stillbirth, itself ‘worse’ than a miscarriage. The loss of a baby with a physical abnormality was also presented as lesser than one which appeared to have no abnormalities. Research on pregnancy loss experienced by surrogate mothers has also noted that different gestational times of loss were ranked as more or less important (Berend, 2012). Pregnancy loss literature therefore has paid some attention to the perceived social value of what has been lost and how such losses can be hierarchised. It also finds that the ambiguity around pregnancy endings provides opportunities for manoeuvre. The lack of certainty and shifting nature of definitional boundaries can leave space for women to propose what has been lost (van der Sijpt, 2020). We also note that some research in psychology queries a link between elapsed gestational time and increased feelings of bereavement (Jaffe & Diamond, 2010; Klier et al., 2002) which is reflected in changing care practices in the English medical context of our recent research projects since Lovell’s early research.

Valuing losses differently connects pregnancy loss to literature in the politics of death, such as work on infant mortality in Brazil in which socio-economic factors and the perceived will to live of undernourished infants affected their mothers’ emotional attachment to them and whether they were grieved (Scheper-Hughes, 1993). Some deaths are socially less valued, to the extent that infant deaths or the deaths of specific racialised groups may not be recorded by the state (Scheper-Hughes, 1996), and the devalued, marginalised, and racialised bodies and tissues may be treated differently in disposal (Denyer Willis, 2018), or in biomedical reuse, experimentation, and storage (Pfeffer, 2009; 2017; Sque et al., 2008). Butler extends this focus on the status of *what or who has died* with their concept, developed in relation to reaction to the 9/11 attacks on the USA, of ‘grievable lives’ in which a ‘hierarchy of grief’ can be perceived (Butler, 2006, p. 32), for example when the American victims of 9/11 were prominently mourned but the victims of US foreign policy in Afghanistan went unnamed. The grievable life notion links the ontological, political, and social status of both that which has died and those who grieve, or do not grieve. These notions of value are connected to Doka’s acknowledgement that some losses such as perinatal death or abortion are defined as insignificant because of what has been lost (Doka, 2002), but they also draw more explicitly on comparison to normative loss, and on relative hierarchies. However, the focus on the emotional responses of ‘grief’ or ‘sadness’ found in literature about value is potentially less useful to some empirical contexts because it excludes other possible responses which are still implicated in hierarchies. In pregnancy ending, ‘grief’ may or may not be the response of those experiencing the event, and in acknowledgement of this we find Robson and Walter’s use of the term ‘loss’ more useful, though we prefer to avoid their generic use of the terms ‘mourners’ and ‘bereaved’.

In this paper, we also draw on the concept of ‘hierarchies of affectedness’ from disaster studies (Andersen, 2013; Brady et al., 2021; Gerster, 2019), itself taking a wider social perspective on Butler’s hierarchies of grief. In hierarchies of affectedness, people in a social setting after a traumatic event assess themselves and one another by comparing and contrasting how they are each affected by the event, and also by other similar events. This results in differentiation, specifically around the negotiated ‘distribution of acknowledgement’ (Andersen, 2013, p. 274). In addition, other gains and losses are perceived to be relevant to the standing of a specific situation – such as the financial gain from insurance of destroyed homes (Andersen, 2013), or government assistance (Gerster, 2019). Using wider contextualisations of loss in this way allows for the identification of further factors contributing to hierarchies of loss, such as the losses of other people, or the consequences of loss. Sometimes contributing hierarchising factors are built into local bureaucracies in the disaster response from authorities (Gerster, 2019). The establishment of hierarchies based on perceived relative affectedness, negotiated around other social factors and events, is an insight from disaster studies which can be fruitfully brought back into analysis of hierarchies of loss.

## Methods

This analysis brings together our separate ethnographic research into women’s experiences of pre-viability pregnancy loss in England. Susie Kilshaw's research has looked broadly at experiences of miscarriage in the UK, including interviews with 40 women who had recently miscarried between 2014-2016 (Kilshaw, 2020; Kilshaw & Borg, 2020). Susie's current research looks at practices around the aftermath of miscarriage including remains disposal. As part of her ongoing ethnographic research in England, she is based at an Early Pregnancy Assessment Unit (EPAU), which sees women up to 16 weeks’ gestation, and the hospital gynaecology ward. Whilst her research includes miscarriages up to 24 weeks viability, the majority of the women she interacts with have experienced miscarriage in the first trimester. Aimee Middlemiss’s research concerns later pregnancy loss, in the second trimester before the legal foetal viability threshold at 24 weeks’ gestation. Aimee’s work considers foetal death, premature labour, and termination of pregnancy for foetal anomaly (TOPFA), all of which are usually managed in the National Health Service (NHS) through the pregnant woman labouring and birthing the foetal body. She conducted ethnographic research in South West England which included interviews with women who had experienced 34 second trimester losses of wanted or accepted pregnancies between 2003 and 2019 (Middlemiss, 2021, 2022) .

Both authors had separately identified data in our research related to hierarchies in pregnancy loss. We brought this data alongside Robson and Walter’s work on hierarchies of loss and the literature reviewed above, and revisited interview data from each of our studies with the hierarchies of loss concept in mind. This produced a new analysis which identified ways of extending the hierarchies of loss concept from this empirical base.

### Analysing Hierarchies of Loss in Pregnancy

The idea that hierarchies of social recognition exist in pregnancy loss was a familiar one to many of our participants, who spontaneously raised issues about the politics of pregnancy loss. We argue that pregnancy loss no longer involves fully disenfranchised grief or silencing in England, where there is increased visibility and social recognition of pregnancy loss through traditional and social media engagement and formal national third sector campaigns such as Babyloss Awareness Week (Sands, 2023) and the National Bereavement Care Pathway for pregnancy loss (National Bereavement Care Pathway, 2022). Instead, we found a more nuanced and complex response based on multiple hierarchies of loss within families, in the wider community, in institutional responses or the response of the state, and even within groups set up to acknowledge pregnancy loss. Such groups are often differentiated by loss classifications – for example, the Miscarriage Association caters for those who experience pre-viability loss, Sands focuses on post-viability loss, and Antenatal Results and Choices (ARC) concentrates on termination for foetal anomaly. The differentiation of pregnancy loss support groups is both useful to women, for example in creating a stigma-free space to discuss termination, and also exclusionary, for example in not recognising similarities between second trimester pregnancy loss and stillbirth. Within groups, some people experienced the production of hierarchies of loss, such as Tamsin,^1^ who lost twins to miscarriage in the second trimester:

It almost becomes a competition. I found that it becomes a competition as to who's had the worst heartache. Who's had it the hardest. And I want to shout at them. And say, ‘you've all been through it!’

Others found explicit recognition of hierarchies of loss to be a basis for challenge and resistance. Helen and her husband joined a support group after their daughter died in the second trimester of pregnancy and became friends with another couple who had experienced two pregnancy losses at later gestations including stillbirth. Helen described how hearing of their losses she felt her own to be less valid:

I said, ‘God, that's hardcore, I feel like what am I moaning about? That's...awful.'

And they were like, ‘Just stop right there. This isn’t comparing about my loss is worse than your loss, or yours is. It’s just not about that.'

And I’ve really learnt since then, it isn’t some sort of league table of pregnancy loss! You just feel like, [in a small voice] ‘mine was only a miscarriage’, and I really stopped myself doing that. Because I’m not belittling my experience.

Some women encountered varied hierarchies as they moved between social worlds. Nila joined a WhatsApp group for experiences of pregnancy loss following fertility treatment after she lost her pregnancy at 7 weeks. Whilst the WhatsApp participants espoused a lack of hierarchy in the face of fertility struggles, comments from staff in medical contexts revealed a hierarchy based on gestational duration:

“Well, you were barely pregnant, what's your point,” that's what it feels like. It’s like, “Well, what does it matter,” it’s as if they think it never existed. Which is hard when after years of trying through IVF, it’s the only pregnancy you managed to have. […] I think I was looking for probably things that the girls had said to me on WhatsApp, to just say, “Of course, this was devastating because this was something you fought for, for so long and you had already.”

Drawing on the many examples of hierarchised pregnancy loss which we found in our combined research, in this paper we identify multiple factors implicated in hierarchies of loss. Following Robson and Walter (2013), we found hierarchies of loss in the degree of perceived closeness of interpersonal relationships to the dead foetal being or baby, which we argue is related to *who is perceived to have lost something* and how this is expressed in social structures or responses. However, in our data hierarchies of loss were also produced in other ways. As Helen’s and Nila’s stories above attest, we found hierarchies built on ontological and institutionalised positions about *what was lost* or died – a foetus? A pregnancy? A baby? We then discuss *what else was lost* besides the deceased, for example plans for children or motherhood, and *the circumstances of loss*. We contextualise loss and its place in hierarchies of loss by showing that it relates to *who else has been lost*, and also *the losses of other people*, touched on in Helen’s story above. We conclude that the expansion of the concept of hierarchies of loss is useful in understanding social constraint and agency in pregnancy and other forms of loss.

### Who is Perceived to Have Lost Something

In pregnancy loss, we concur with Robson and Walter (2013) that perceived closeness of relationship to the loss is a factor in whether the loss is recognised by others. Pregnant women, in relation to whose bodies the loss is directly experienced, are broadly considered to be those who have the most intense experience of loss. Any supportive response from family and friends tends to be primarily offered to the woman, and institutional support offered by the NHS after loss tended to focus on the pregnant woman, with exceptions in some NHS settings. For example, Alice was offered counselling for her grief after termination for foetal anomaly, but the administrative staff refused to include her husband, who also considered himself to have lost a baby. Although male or non-pregnant partners are usually understood to be the next most affected person in pregnancy loss, they are often assumed by others to be experiencing a less intense reaction (Lang et al., 2011; McCreight, 2004). Any loss felt by other family members, such as siblings or grandparents, was generally not acknowledged by people outside the family, as other literature in perinatal loss has noted (Murphy & Jones, 2014). Normative ideas about hierarchies of affectedness can be embedded in institutional responses (Gerster, 2019), in this case derived from assumptions about relational closeness to the object of loss which were not shared by the persons experiencing loss.

At the same time, other women wanted normative relational hierarchies to be maintained. Angela felt her own mother should have supported her, as the main mourner, rather than felt the loss so acutely herself:

My mum was devastated. Again, my third pregnancy, her only daughter. […] She didn’t deal with it very well and she didn’t help me at all. It really, I found that very difficult. Your mum. It’s the one person you just kind of want to…you know, even though I was 40, I wanted my mum to put her arms around me. But she never did that. It was just too traumatic for her.

For Charlie and Kerry, their partners’ response to the death of their babies in the second trimester was similarly insufficient. Charlie described how her then partner, the biological father of the baby girl who died, never expressed grief or visited her grave. Kerry was hurt by her partner’s lack of public acknowledgement of the baby boy who died as his only son. For these women, a normative hierarchy of loss meant that the biological fathers of the babies *should* have displayed more emotion, and the absence of this was threatening to their own place in the hierarchy of loss as the corresponding biological mother. For many women, lack of normative response which they understood to be appropriate to the hierarchy of loss in which they placed themselves also threatened the status of the being which had died. In these cases, women sought to strengthen hierarchies based on normative relations to the deceased.

### What was Lost

In pregnancy loss, we found that the most important factor in the production of hierarchies of loss was the status of the dead foetal being or baby. As in Helen, Tamsin and Nila’s experiences recounted above, and similar to findings from the early 1980s (Lovell, 1983) and in other contexts (Cassidy, 2021), the gestational timeframe at which the loss occurred defined its presumed gravity, whether it was socially legitimated, and the distribution of acknowledgement. Generally, the greater the gestational age of the foetus, the more the loss was liable for recognition. This scalar hierarchy is emphasized by the UK’s legal foetal viability threshold of 24 weeks’ gestation, after which a pregnancy loss is legally defined as a stillbirth. Prior to 24 weeks, the being which was lost is legally an embryo, foetus, pregnancy, part of the pregnant woman, whereas after 24 weeks it is a stillborn baby acknowledged by the state on the Stillbirth Register (Middlemiss, 2021). The gestational timeframe intersects with the legal position on live birth, where a baby medically confirmed alive at birth who then dies must registered as a birth and death at any stage in pregnancy. Some beings which die during an event of pregnancy loss are legally understood to be full persons, others are understood to be stillborn babies, and others are not considered to be separate beings from the pregnant woman. There are institutional and bureaucratic consequences of the categorisations such as access to maternity pay and forms of employment leave, the type of medical treatment available to women (Middlemiss, 2022), and choices about disposal of the body (Middlemiss, 2021). These institutionalised legal positions map onto normative hierarchies of loss which assume different degrees of grief, as expressed by Chloe shortly after her daughter died at 18 weeks’ gestation:

I kept thinking as well, I actually did say this, ‘but at least she didn't die in childbirth, at least it wasn't like a stillbirth at 39 or 40 weeks.' And because in my, it's almost like I'm thinking to myself that would be worst. […] And you hear about neonatal death in the first 4 weeks - oh my god, imagine that, when you've held them. You know.

Women in our research both accepted and challenged these hierarchies of loss based on the status of the foetal being. Simone’s fourth child died *in utero* in the second trimester:

I know people that have lost, like, babies, before 12 weeks and they said to me, ‘Oh but it wasn't as bad as you.'

And I’ve always said to them, ‘No, but it was bad for you. Like, don’t compare yourself to me, it was still bad for you.'

And then I met this mum at a group and she’d lost her baby at 42 weeks. And I hear myself saying the same thing: ‘Oh, my loss wasn’t as bad as yours.'

The simultaneous acceptance that losing a foetal being at a later stage is ‘worse’ and yet an earlier loss is equally valid was a common theme in the research. For some women, it became important to emphasize gestational duration over foetal gestational age, because in cases of foetal death the pregnancy may extend beyond the death until it is discovered, as Mia explained:

Like that somehow this needs to matter. So, I think, yes, I’ve noticed that that I do stress that it was nine weeks and that it was discovered in my eleventh week… That always seems to be a reference for me.

Mia could express the significance of her loss by emphasizing her pregnancy duration rather than foetal gestational age, agentially adapting the hierarchy of loss whilst still aligning herself with hierarchies of recognition.

A further factor in producing hierarchies of loss was the degree of formation of the foetal body, which is linked to gestational foetal age. For women who had experienced both early and late losses in their reproductive lives, the more developed foetal bodies were felt to be more significant and impactful losses. Women spoke about thresholds or experiences in their pregnancy linked to the developing foetal body, which they saw as informing a scale of loss. Having a ultrasound scan and observing the active foetal body, hearing a heartbeat, feeling foetal movement, and learning foetal sex were all described as interactions that contributed to the gravity of loss and therefore placed it in the hierarchy of pregnancy loss. Some women experiencing earlier losses perceived their experience as less significant if there was an absence of these plot points. At times, they did so to explain feeling less grief or distress than that which might be expected of them. Nicole’s missed miscarriage was discovered at her routine 12-week scan. Had the pregnancy been ongoing, this would have been her first opportunity to see the foetus, but instead she was told the pregnancy had stopped developing at a very early stage and there was no visible foetal body. For Nicole the fact that there was “no baby to grieve” was a comfort, a “way of coping and moving past the experience”. She emphasised foetal development, or its lack, rather than pregnancy duration to explain her reaction to the loss, whereas Mia emphasised the duration of her pregnancy rather that the gestational age of the foetus to lend gravity to her sense of loss. Similarly, Samantha was sad for what could have been, but didn’t grieve the loss of a concrete being because she did not witness a foetal body:

I’m sad that this happened but I don’t feel like I’m attached to it- I haven’t seen that this has a heartbeat or is a life yet. It’s just a thing that I was excited about and now I can’t have, yet… rather than a real person or a real being....It wasn’t a baby yet because I hadn’t seen it with a heartbeat or felt it.... I think if I’d seen a heartbeat, if I’d had a scan and then it had happened afterwards, I think it would be really different.

The value of the foetal being as an object of loss was also hierarchised in relation to its perceived physical normativity. A common response from other people to miscarriage was to say that the foetal being ‘must have had something wrong with it’, with the implication that the death was for the best and that grief should be attenuated. This was how Paula felt, taking her termination for foetal anomaly as the loss of a pregnancy and possible family future but not the loss of a baby. She found the positioning of the event as a tragedy by medical staff to be jarring:

I didn't want to really hear that. That sympathy. Because by that point, I'd actually gone so clinical that I wasn't even recognising it as a baby or a foetus, it was just something that I needed to get rid of.

Similar conflicting hierarchies were felt by some women experiencing early pregnancy loss when confronted with discussions about the disposal of pregnancy tissue. As (Kuberska, 2020) has shown and Susie’s ongoing research reveals, UK practices around disposal of pregnancy remains typically afford them the same treatment as would be expected of a later term loss or miscarriage. Yet this did not always accord with women’s wishes, as Beth explained:

One of the ways I had been coping with the whole miscarriage, it was very early, I was maybe six or seven weeks. For me, at that point… is it’s not a baby. It’s a clump of cells that has a lot of hopes and dreams attached to it and it could be a baby, but it’s not a baby … So then being confronted with the question of that consent form [for disposal] and the treating of it as more than a small clump of cells, was just, it made it a lot more upsetting because it makes it a baby, which isn’t how I had been thinking of it.

Institutional practices around disposal had the effect of constructing Beth’s pregnancy end as the loss of a person or baby, which conflicted with her framing of it.

By contrast, Tess felt her daughter who died through termination for foetal anomaly was her child, but she found that other people responded with a shudder to images of the baby, and some family refused to attend the funeral. For women like Tess, the hierarchy which said their baby was less worthy of grief was distressing and was resisted by action asserting value as a person. For other women, the hierarchy of value based in the normative body added to their distress because their baby appeared to be normal, and therefore they could not understand the death. Phoebe’s son died during premature labour and she speculated that if he had had a foetal anomaly she would have been more able to accept his death because this accorded with the hierarchised value she would have placed on an abnormal foetus. Ontologies of pregnancy and the foetal being as the loss objects were thus fundamental to the position of the loss within hierarchies of loss for women and for other people. Where the loss was perceived to be that of a full person or baby, it carried more weight than the loss of an early pregnancy or foetus. If the foetal being was considered damaged or lacking in some manner, then it could potentially be lower down the hierarchy of loss.

### What else was Lost

Our data also showed that beside the loss of the pregnancy, foetus, or baby, women experienced secondary losses associated with, but distinct from, the primary object of loss. Literature from pregnancy loss studies has described early miscarriage as the loss of possibility (Frost et al., 2007), and as the loss of the social role of motherhood (Layne, 2003). These losses were present in our research, for example when Louise said of her first pregnancy ending in miscarriage: ‘when you lose one child you’re like, not a mother, you’re a nothing?'. For some women, an expected normative life course (Becker, 1999) was also lost, such as when Georgia contrasted her miscarriage with her friends' new babies. Secondary factors could move the original loss up the hierarchy of loss for the women experiencing it. The effort which had gone into achieving pregnancy at all was also relevant – Holly’s polycystic ovaries meant it had taken three years to get pregnant and then her daughter died. Mia, Nila and Angela all became pregnant through repeated rounds of IVF, only to lose their pregnancies. Loss became entangled with experiences of fertility uncertainty, as Beth articulated:

I think what makes it potentially more difficult than maybe it is obviously, because we’ve got all the fertility problems tied in with it. So, it kind of is all quite enmeshed together. So it’s sort of am I upset about the miscarriage or am I upset about the fact that I’m still not pregnant?

Grace’s loss was compounded by anxiety about age and a closing fertility window meaning the possible end of her reproductive journey when she lost her 8 week pregnancy conceived after her 40^th^ birthday. Some women linked these difficulties to their gender identity and felt their womanhood was threatened by the secondary loss of infertility, as noted in stillbirth research (Murphy, 2019).

For others, a secondary loss was that of security and trust in either biomedicine or future pregnancies. Many women, including Angela, Eva and Helen, had traumatic medical experiences related to their pregnancy loss, requiring hospitalisation, high dependency care, or blood transfusions. For some, such as Gemma, the loss experience contributed to a decision to have no further children. For those who went on to be pregnant again, subsequent pregnancies were experienced as highly anxious. Women spoke about an associated loss of the experience of pregnancy as a happy, celebratory time. Such secondary losses contributed to the impact and gravity of pregnancy loss.

### The Circumstances of Loss

A politics of intentionality hierarchised pregnancy loss for our participants who had experienced termination for foetal anomaly. As in Denmark (Heinsen, 2022), there was ambiguity in relation to what had been lost and the social expectations around whether and how to grieve. Paula and her husband, as noted above, felt that grief was inappropriate. Other women who mourned their terminations felt that because they had ‘chosen’ to end the pregnancy others perceived the event as self-induced and therefore less serious. Amber felt stigma about termination meant other people would not accept her grief for her baby who died:

I could say with my first miscarriage that I lost it. The baby didn't survive. But with this, it was through my action. So it's a hard one. I...Once it happened, I wanted everyone to know, and no-one to know. I couldn't look people in the eye. I felt really ashamed.

This hierarchisation related to the intentionality read into termination was expressed by some other women in the research who lost their pregnancies spontaneously and wished to make a distinction between their experience and that of abortion. Charlie lost two babies spontaneously and described how she discussed her losses with her friend who had had a termination for foetal anomaly:

I said, 'I could never do that, personally,' like, we're friends, I thought we could have the conversation. I'll support her, for what she's going through, but I said to her 'it's not something I could ever do.'

She was like, ‘But it’s just the same.'

I said, ‘You’re trying to play God.'

And then she said ‘But it’s the same, because you go into a pregnancy knowing you could lose them.’

And I said, ‘No, I go into pregnancy knowing I’m going to fight everything I can to keep them.'

Such hierarchies meant women who experienced termination for foetal anomaly were cautious about disclosing their loss, as Lucy explained:

You really have to kind consider what information you give to whom, because you’re never really sure…how it’s going to go. Which leaves you feeling…a bit sort of…makes it feel taboo, makes it feel restricted. […] I kind of feel like of all the baby loss, ours is at the lower spectrum of acceptability?

Pregnancy endings were also situated in relation to one another. Alice experienced two terminations, for different congenital foetal anomalies. She described herself as still somewhat ambivalent about the second termination, because of a perception of the foetal anomaly as less serious, whereas the first termination was for a condition incompatible with life. As a result, she presented the second loss to many people as spontaneous. Ruth experienced miscarriage after deciding to terminate her pregnancy and felt she could not disclose the miscarriage because the sympathy she would receive would not have been offered if she had terminated the pregnancy. Whilst she had planned to memorialise the pregnancy ending through termination, once miscarried she found she no longer wanted to mark the loss:

I would have been potentially putting an end to a life that was viable so I suppose it would be mourning the potential. Whereas with a miscarriage there wasn’t potential. It didn’t, it sort of petered out… of its own accord.

The circumstances in which loss happened and the degree of perceived intentionality and responsibility was a factor in a hierarchised politics of loss sometimes challenged, sometimes accepted.

### Who Else has Been Lost

Hierarchies of loss were also produced by comparison with other, non-pregnancy deaths. People drew on other deaths they had experienced to explain why the pregnancy loss was ‘worse’ or more impactful for them. Louise went through a termination for foetal anomaly before her father-in-law died unexpectedly:

We had [father-in-law’s] funeral, about two weeks after. That was horrendous. We had to go. And I was quite angry with people then. Because, like, he was 82 and he had a life. Not, I dunno, not angry. But even [husband] could see that perspective. Cos we'd just lost a child. At the start of life. […]

I felt, what about my grief? What about what I’ve just been through? I couldn’t relate.

Louise felt the social acknowledgement of the older man’s death to be threatening to her own loss, in the context of ideas of partial personhood in the unborn baby. She contrasted the idea of a completed life with the idea of a dead baby, to evoke a hierarchy of loss. Bethany’s beloved cousin died through suicide at a young age, and she connects her grief with what she felt a few years later when her son died at 17 weeks of pregnancy:

I've lost older relatives too, but...and that's obviously like...sad. But it's not the same? That's totally different, that's like...you kind of expect that don't you? That's kind of how things go.

Youth, and the unexpectedness of suicide and pregnancy loss moved these losses up the hierarchy of comparative loss, even as they might not be acknowledged by others. By contrast, Simone, whose fourth child died in pregnancy, had recently lost her mother. She felt the comparison made by close family between the grief which was acceptable for a mother and the grief which was acceptable for an unborn baby meant she had to suppress the intensity of her pregnancy loss grief:

I remember at the time I just felt I was still grieving my mum, and now this. The only way I can describe it is I was overflowing with sadness. […] But yeah, I think because of what happened to my mum, I kind of had to get over the baby a bit quicker? Because obviously my mum was worse, she was an actual person. And as far as people were concerned, I was ok with that, you know…

A specific death can therefore produce hierarchies of loss in relation to other deaths, either to value this death as the ultimate loss, or to devalue it in relation to deaths perceived as more important. These comparative hierarchies also echo the contrasts discussed above in relation to multiple pregnancy losses experienced at different gestational times. Women both produced their own hierarchies, and experienced hierarchisation by other people.

### The Losses of Others

Not only are other deaths which have been personally experienced factors in the production of hierarchies of loss, but so are losses experienced by other people. Limitations are placed on the acknowledgement of loss and grief in relation to what other people have lost. This can be seen above in the distinctions made between termination for foetal anomaly and spontaneous loss, in the distinctions between pregnancies lost at different gestational stages, and confounding factors such as IVF. These comparisons bear similarities to hierarchies of affectedness in disaster studies (Andersen, 2013; Brady et al., 2021; Gerster, 2019), in which acknowledgement is differently distributed because some people are thought to suffer more direct or extensive loss, or some disasters outweigh others in scope or scale. Eva experienced losses in the first and second trimesters, and the latter experience left her unable to accept the support of an acquaintance in her village:

She’s had a few miscarriages, at one point you know, she really wanted to bond with me over [son who died] I suppose. But she had an early miscarriage and I suppose in my head I just couldn't relate to what she'd gone through compared to what I'd gone through. […] I felt I couldn't really relate, because I was like, you haven't given birth to a baby on the loo.

Such scalar assessments of pregnancy loss compared to the losses of others could also make women feel unentitled to grieve. Simone had a relative who never had a living child, and felt guilty that her own daughter’s death was constantly on her mind:

I feel like it shouldn't be, because I've got [new baby girl born since the loss]. [crying] Because, do you know, like, when people lose a baby or whatever and then they can't have any more, I just feel like they're the ones that...should have the sympathy and the understanding. Not me that went on to have my baby girl. I feel like I haven't got the right. […] At least I went on to have another one, I've still got three. Because that was the other thing people told me, ‘At least you've still got your other three children.'

Losses were understood to be somewhat mitigated by circumstances such as already being a mother, or having subsequent children, and this moved them down a hierarchy of perceived affectedness.

## Conclusion

In this paper, we have shown that hierarchies of loss are perceived, experienced, made, and resisted in circumstances of pre-viability pregnancy loss in England. As well as being an empirical contribution to the field of pregnancy loss research, the paper also contributes to wider understandings of the politics of loss, death, and grief by extending the notion of hierarchies of loss. Previous literature drew attention to loss which is socially unrecognised through the concept of disenfranchised grief (Doka, 1989), but this took for granted the ontological status of what was lost as a grievable object and could not pay attention to the foetal being as a complex and mutable object of loss. Disenfranchised grief, as Robson and Walter (2013) showed, assumed a binary of enfranchisement or disenfranchisement which no longer applies to pregnancy loss in England. The concept also assumed a passivity in the disenfranchised mourner which does not allow for the agency and resistance found in some women’s response to pregnancy loss.

Robson and Walter (2013) challenged the disenfranchised grief binary through a relational analysis of degree and scale of loss, but they also considered the object of loss to be a given, stable entity without the capacity to influence relationality. Drawing on literature in the politics of death which considers the value of what was lost, we have shown above how aspects of the foetal body such as gestational time and normative formation are factors influencing hierarchies of pregnancy loss in relation to ontological positions on what was lost. Through our empirical data, we have demonstrated that hierarchies of loss are experienced and produced by further relational factors besides the relationship to the deceased, including other losses and the losses of others. Attention to the institutional, bureaucratic and normative consequences of hierarchies of loss is also a way of understanding their effects in the world. Relating hierarchies of pregnancy loss to hierarchies of affectedness (Andersen, 2013) further extends the two concepts.

Finally, this paper demonstrates the breadth and variety of hierarchies of loss as tools in the politics of death and grief which are actively used to make claims, to silence or exclude, to allocate and ration social resources. In the context of pregnancy loss, women do experience hierarchies as social constraint, in some cases as disenfranchised grief. However, they also recognise and use hierarchies of loss in agential ways, sometimes to convey the impact and bolster the social status of their own loss, sometimes to assert the legitimacy of the foetal being or baby that died, sometimes in seeking insight into their experiences. They may actively contest and resist hierarchies of loss from other people, or offer solidarity to others in defiance of normative hierarchies. This insight from pregnancy loss gives a dynamism to the concept of hierarchies of loss which can account for change and political action, and can make visible nuanced politics of loss. It extends the concept of hierarchies of loss so it can include experiences which do and do not involve grief and bereavement, and experiences of social recognition alongside those where loss is disenfranchised, marginalised, or ungrievable.

## Note

1. Participants have been pseudonymised in the text where they requested this.
